# Recovery and Separation of Dysprosium from Waste Neodymium Magnets through Cyphos IL 104 Extraction

**DOI:** 10.3390/ma15155281

**Published:** 2022-07-30

**Authors:** Wei-Sheng Chen, Guo-Cai Jian, Cheng-Han Lee

**Affiliations:** Department of Resources Engineering, National Cheng Kung University, No. 1, Daxue Rd., East Dist., Tainan City 701401, Taiwan; kenchen@mail.ncku.edu.tw

**Keywords:** rare earth elements, recovery, neodymium magnet, Cyphos IL 104, ionic liquid

## Abstract

In the present study, the extraction of rare earth elements (REEs) from waste neodymium magnets using phosphorus ionic liquid Cyphos IL 104 was investigated. The objective was to recover and separate the heavy REE (Dy) from light REEs (Nd and Pr). Therefore, the experimental parameters of ionic liquid extraction, including contacting time, the initial pH value, extractant concentration, and O/A ratio, have been optimized. The highest separation factor α_Dy/Nd_ of 45.18 and α_Dy/Pr_ of 47.93 has been achieved. Following the ionic liquid extraction, the comparison of different stripping agents and the stripping parameters (the concentration of stripping agent and A/O ratio) were also explored. In short terms, this research demonstrates the optimal parameters of Cyphos IL 104 for selectively extracting high REE (Dy) and reveals its potential for recovering and separating REEs in real waste.

## 1. Introduction

In recent years, rare earth elements (REEs) have become essential ingredients for modern technology as they are widely used in electric vehicle batteries, electronics, powerful magnets of devices and wind turbines, phosphors of fluorescent lamps, catalysts of petroleum refining industries, laser products, etc. [1,2]. That is why REEs are called the vitamins of modern industry. However, due to the uneven distribution of REE reserves as well as the environmental impact during their production [3,4], there are only a few countries that have mine production of REEs. In 2020, as the world’s largest rare earth producer by far, China accounted for a 57.6 percent share of the total global rare earth mine production, according to the data from the U.S. Geological Survey [5]. 

Among the REE-containing products, the NdFeB permanent magnets, containing neodymium, praseodymium, and dysprosium, are the most widely used in related industries, which account for at least a quarter of REE products [6,7]. The magnets perform well in the industries because they have high magnetic fields and high resistance against demagnetization, which allowed them to be widely applied in computer hard disk drives, loudspeakers, headphones, and electric motors in vehicles or wind turbines [1,6,8,9]. Considering the wide application of neodymium (NdFeB) magnets and their growing demand on the global market, rare earth elements like neodymium (Nd) and dysprosium (Dy) have been listed as critical metals with high supply risks by Taiwan, Korea, Japan, America, and the EU. In addition, with the advancement of technologies and reduction of product life cycles, there is an increasing number of secondary resources like neodymium magnets that could be recycled annually [10,11,12,13]. Therefore, it is economically and strategically necessary to develop recovery technology for waste neodymium magnets.

In the present study, the solvent extraction process in hydrometallurgy was applied, which is the most common method used to recover and separate REEs [14,15,16,17,18,19]. The solvent extraction process applies two immiscible liquids representing the organic phase and aqueous phase. The two phases were shaken and mixed in the separatory funnel when the target metal ions were extracted to the organic phase and separated from other elements because of their different affinities [20,21]. Compared to traditional organic extractants, the organic phase in this study is a phosphorus ionic liquid (IL), Cyphos IL 104 (trihexyl(tetradecyl) phosphonium bis(2,4,4-trimethylpentyl) phosphinate). There are two main advantages of applying IL to the solvent extraction process [20,21,22,23,24]. On one hand, the functional ionic group could be easily recomposed because users need to convert the affinity and selectivity with metals, which is why it is called a designer solvent. On the other hand, IL is considered a green solvent because of its low volatility and high thermal stability, making it easy to be regenerated and reused in the process and also reducing the wasted chemicals. This is the most attractive characteristic of ILs in view of the prevention of secondary pollution and a reduction in the costs of chemicals used in the whole process.

There has already been some literature demonstrating the extraction of metals through ionic liquids. The imidazolium ionic liquids ([C_n_mim] where n = 2, 4, 6, 8, 10) were first introduced into the metal extraction process [25,26,27,28,29]. For example, the separation of Ce^4+^ from La^3+^ and Th^4+^ applying [C_8_mim][PF_6_] has been investigated before [30]. Synergistic extraction effects of [C_4_mim][NTf_2_] and [C_2_mim][NTf_2_] with four conventional extractants to separate Pr^3+^, Gd^3+^, and Dy^3+^ were also studied by other authors [31]. The phosphonium ionic liquids, [C101][SCN] and [C101][NO_3_], which were derived from trihexyl(tetradecyl)phosphonium chloride (Cyphos IL 101), have been used to separate Nd^3+^ and Dy^3+^ with and without the addition of Cyanex 923 [32]. Additionally, Cyphos IL 104 has shown the ability to extract Nd^3+^ and separate trivalent lanthanides from each other [33]. In addition, extraction involving Cyphos IL 104 has also been successfully conducted on other metals [34,35,36,37,38,39]. These studies indicated that the properties of low vapor pressure, high thermal stability, and the high extraction efficiency of Cyphos IL 104 could overcome the shortcoming of conventional organic extractants. To realize the selectivity of REEs through solvent extraction and IL extraction, the comparison of separation factors is shown in Table 1.

In the present study, Cyphos IL 104 was applied on the recovery of real waste neodymium magnets that have not yet been explored. The aim was to recover and selectively extract the heavy REE (Dy) from light REEs (Nd and Pr). Thus, the experimental parameters of ionic liquid extraction, including the contacting time, the initial pH value, the extractant concentration, and the organic/aqueous volume ratio (O/A ratio), were optimized in this study. Furthermore, the comparison of different stripping agents and the stripping parameters (the concentration of stripping agent and the A/O ratio) were also investigated after the extraction process.

## 2. Materials and Methods

### 2.1. Reagents and Chemicals

**Figure 1:**
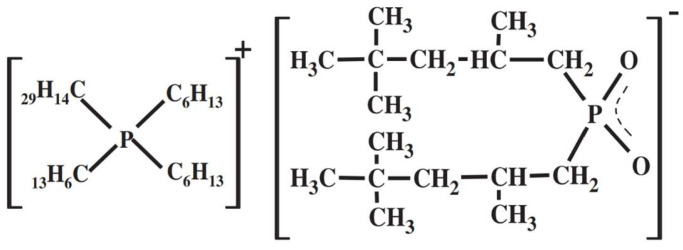
The structure formula of Cyphos IL 104 [R4PA].

Cyphos IL 104 from Sigma Aldrich (St. Louis, MO, USA) is a phosphonium-based and hydrophobic ionic liquid (≥90%, water <1%) that was used as an extractant, and its structure is shown in Figure 1 [33]. As the organic diluent of Cyphos IL 104, reagent grade kerosene was supplied by CPC Corporation (Kaohsiung, Taiwan). Aqua regia used for the complete leaching was prepared by mixing nitric acid (≥65%) and hydrochloric acid (≥37%), both acquired from Sigma-Aldrich (St. Louis, MO, USA), in a 1:3 volume ratio. Nitric acid (≥65%) and ammonia (30–33%) were acquired from Sigma-Aldrich (St. Louis, MO, USA). They were used to adjust the pH value in the precipitation and extraction process. In the stripping process, hydrochloric acid (≥37%), nitric acid (≥65%), sulfuric acid (≥98%), ammonia (30–33%), and oxalic acid (≥98%) were all obtained from Sigma Aldrich (St. Louis, MO, USA) to strip dysprosium back to the liquid phase. During the analysis procedure, ICP standard solutions of all elements were acquired from High-Purity Standards, Inc. (North Charleston, SC, USA). The nitric acid (≥65%) was purchased from Sigma-Aldrich (St. Louis, MO, USA) and diluted to 2% to be the background value and thinner for ICP analysis. All chemicals were analytical grade and used without further purification. In addition, all chemicals and aqueous solutions were diluted by deionized water (resistivity 18.0 MΩ·cm) to avoid impurities affecting the results.

### 2.2. Apparatus

The demagnetization was conducted by a muffle furnace (LE 6/11, Naberthem, Lilienthal, Germany). The concentration of metal ions in solution was analyzed by inductively coupled plasma optical emission spectrometry (ICP-OES; Varian, Vista-MPX, PerkinElmer, Waltham, MA, USA). The pH value was measured by a pH meter (SP-2300; SUNTEX; New Taipei City, Taiwan). The relative standard deviation (RSD) of ICP-OES and the pH meter were below 3% and 1%, respectively. The solvent extraction process was accomplished using a thermo mixer incubator (TMI-100H; ChromTech, Apple Valley, CA, USA) to mix the organic phase and aqueous phase in a separatory funnel and maintain the temperature.

### 2.3. Pretreatment and Complete Dissolving

The NdFeB magnets from end-of-life motors of electric vehicles were provided by a local recycling company. The demagnetization of waste magnets was conducted in a muffle furnace by heating to 600 °C for 3 h at a heating rate of 10 °C/min. After the demagnetization, the magnets were manually crushed and ground to particles. One gram of the particles was then completely dissolved by 120 mL aqua regia at 80 °C for 24 h. After filtration of the total dissolution (ADVANTEC cellulose acetate membrane filter with pore size 0.45 μm), no residue was observed, and the filtrate was then diluted to 1 L for the elemental analysis and following experiments.

### 2.4. Ionic Liquid Extraction

In this study, Cyphos IL 104 as an extractant was diluted into kerosene to extract rare earth ions from the solution. To determine the affinity of Cyphos IL 104 with different metal ions, distribution ratio (D) and extraction efficiency (E) were used in this research. D and E were correlated with the concentration ratio of one metal in the organic phase and the aqueous phase at equilibrium and can be written as Equations (1) and (2), respectively:

(1) $$D_{A}=\frac{{\left[A\right]}_{\mathrm{org}}}{{\left[A\right]}_{\mathrm{aq}}}$$

(2) $$E_{A}\left(\%\right)=\frac{{\left[A\right]}_{\mathrm{org}}}{{\left[A\right]}_{\mathrm{aq}}+{\left[A\right]}_{\mathrm{org}}}\times 100$$

where [A]_org_ and [A]_aq_ are the concentrations of one metal (A) in the organic and aqueous phases at equilibrium.

The separation factor (α) was introduced to determine the selectivity of the extractant on one metal (A) over another metal (B) and can be written as Equation (3):

(3) $$\alpha_{A/B}=\frac{D_{A}}{D_{B}}$$

After the extraction process, the stripping process was conducted to get rare earth ions back to the liquid phase, and the stripping efficiency is represented as Equation (4):

(4) $$S_{A}\left(\%\right)=\frac{{\left[A\right]}_{\mathrm{aq}}}{{\left[A\right]}_{\mathrm{aq}}+{\left[A\right]}_{\mathrm{org}}}\times 100$$

## 3. Results and Discussion

### 3.1. Elemental Analysis

After being demagnetized and ground, the magnet powder was dissolved by aqua regia and its composition was measured by ICP-OES. Table 2 shows the elemental analysis results in weight percent. As the major component, iron accounts for 64.62%. As for the target metals, the light rare earth elements Nd and Pr and the heavy rare earth element Dy account for 20.73%, 5.08%, and 3.55%, respectively.

To remove the iron from the solution, we added diluted ammonium hydroxide to adjust the pH value to more than 3.5 and left it at room temperature for 18 h. After separating the filtrate from the residue and element analysis, we found that iron completely precipitated with less than 2% coprecipitation of rare earth elements, which means Nd, Pr, and Dy became the major components of the solution, as shown in Table 3.

### 3.2. Extraction

#### 3.2.1. Effect of Contacting Time

**Figure 2:**
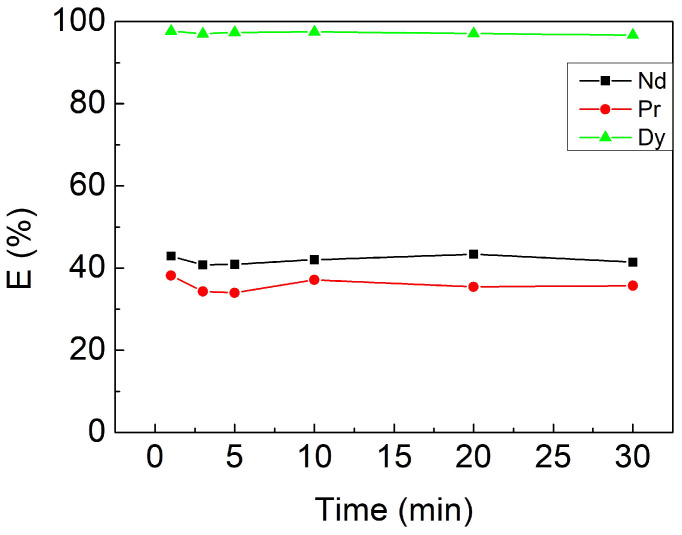
Extraction efficiencies at different contacting times. Fixed parameters: pH 5, 3 mM [Cyphos IL 104], O/A ratio of 1.

Because the other minor components, such as Co, B, Ni, and Cu, were negligibly extracted, only REEs were discussed in this research. In this section, the equilibrium contacting time needed for the extraction process was investigated, which was controlled by adjusting the separatory funnel shaking period from 1 to 30 min. Other initially fixed parameters were pH 5, 3 mM [Cyphos IL 104], and an O/A ratio of 1. As shown in Figure 2, the reaction rate of Cyphos IL 104 extracting rare earth elements is rarely fast, which reaches equilibrium in 1 min. The results are in accordance with previous studies applying Cyphos IL 104 to the extraction of other metals [35,36]. To ensure the complete reaction, the contacting time of 10 min was chosen as the optimal parameter.

#### 3.2.2. Effect of Initial pH Value

**Figure 3:**
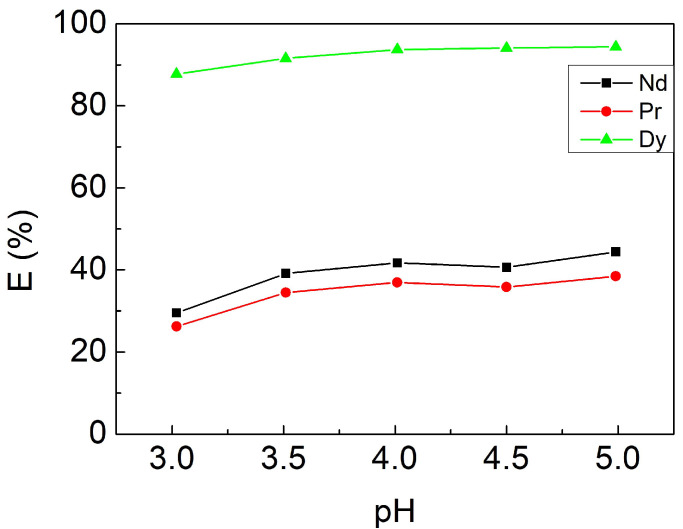
Extraction efficiencies at different initial pH values. Fixed parameters: contacting time of 10 min, 3 mM [Cyphos IL 104], O/A ratio of 1.

Since the extraction efficiency of Cyphos IL 104 is pH value-dependent, the reaction at different pH environments was investigated by adjusting the initial pH values from 3 to 5. The other fixed parameters were a contacting time of 10 min, 3 mM [Cyphos IL 104], and an O/A ratio of 1. The results shown in Figure 3 indicate that the extraction efficiency of rare earth elements slightly increased with increasing pH value. The priority of Cyphos IL 104 to combine with hydrogen ions over the rare earth metals ions might be the reason. The reaction mechanisms have been proposed [33,40] and shown in Equations (5) and (6), where RE represents the rare earth elements. Therefore, the reaction between Cyphos IL 104 and RE metals ions was weaker in a lower pH value environment. Nevertheless, pH value 3 was chosen as the optimal parameter for the following experiments because the purpose of the present study was to separate the heavy REE from the light REE.

R_4_PA_org_ +H^+^_aq_ + Cl^−^ _aq_ → R_4_PCl_org_ + HA_org_ (5)

RE^3+^_aq_ + 3Cl^−^_aq_ + 3R_4_PA_org_ → 3R_4_PCl_org_ + REA_3org_ (6)

#### 3.2.3. Effect of Cyphos IL 104 Concentration

**Figure 4:**
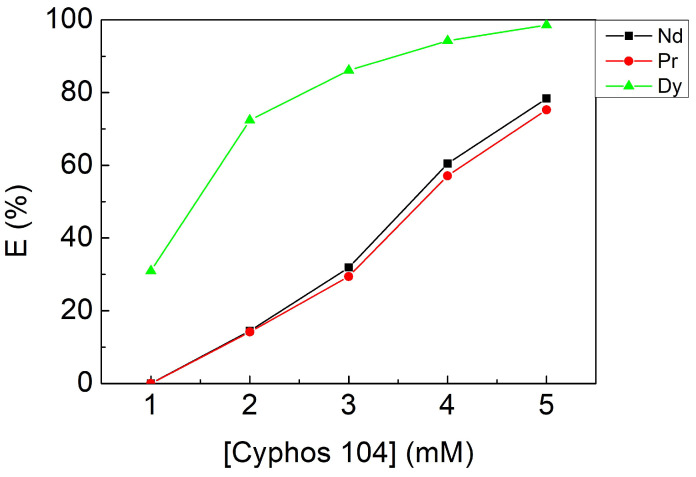
Extraction efficiencies at different Cyphos IL 104 concentrations. Fixed parameters: contacting time of 10 min, pH 3, O/A ratio of 1.

To investigate the effect of extractant concentration, the concentration of the Cyphos IL 104 and kerosene system was changed from 1 to 5 mM with the fixed parameters: a contacting time of 10 min, pH 3, and an O/A ratio of 1. Figure 4 shows that the extraction efficiency increased along with increasing Cyphos IL 104 concentration as the equilibrium of Equation (6) shifted to the right-hand side. However, 2 mM was chosen as an optimal parameter because of the high extraction efficiency and selectivity where α_Dy/Nd_ and α_Dy/Pr_ equaled 20.30 and 28.89, respectively.

#### 3.2.4. Effect of O/A Ratio

**Figure 5:**
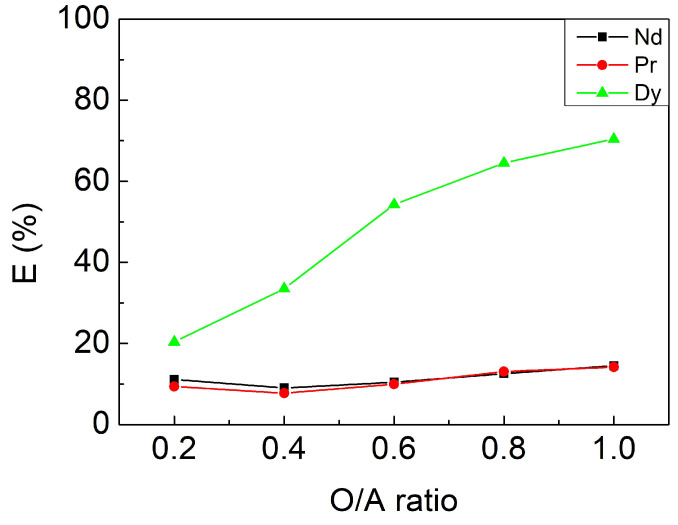
Extraction efficiencies at different O/A ratios. Fixed parameters: contacting time of 10 min, pH 3, 2 mM (Cyphos IL 104).

To study the maximum loading of the organic phase per unit volume, the organic to aqueous phase volume ratio was adjusted from 0.2 to 1 (mL/mL) with fixed parameters: a contacting time of 10 min, pH 3, and 2 mM [Cyphos IL 104] as prior results. Figure 5 reveals that the highest separation factor α_Dy/Nd_ of 45.18 and α_Dy/Pr_ of 47.93 was achieved when the O/A ratio was 0.6, where E_Dy_, E_Nd_, and E_Pr_ were 54.30%, 10.44%, and 9.94% respectively. As a result, the O/A ratio of 0.6 was chosen as the optimal parameter for the extraction experiment, which can reduce the dosage of extractant and also increase the element concentration in the organic phase.

In brief summary, we derived the optimal parameters for extraction with a contacting time of 10 min, pH 3, 2 mM [Cyphos IL 104], and an O/A ratio of 0.6. The separation factor α_Dy/Nd_ of 45.18 and α_Dy/Pr_ of 47.93 was achieved, where E_Dy_, E_Nd_, and E_Pr_ were 54.30%, 10.44%, and 9.94% respectively. Additionally, four-stage extraction cycles can be conducted to acquire more than 95% Dy from the aqueous phase.

### 3.3. Stripping

#### 3.3.1. Stripping Agent

To compare the stripping efficiencies of different chemicals, the commonly applied stripping agent including sulfuric acid, nitric acid, hydrochloric acid, oxalic acid, and ammonium hydroxide were individually used in the stripping process. The fixed parameters were a concentration of 1 M, an A/O ratio of 1, and a contacting time of 10 min. Table 4 demonstrates that oxalic acid and ammonium have lower and limited stripping efficiencies, respectively, while the three strong inorganic acids, sulfuric acid, nitric acid, and hydrochloric acid, showed the ability to strip back the RE ions from the organic phase. Among them, nitric acid was the chemical with the highest selectivity of heavy REE over the light REEs. Therefore, nitric acid was chosen as the stripping agent to optimize the following parameters.

#### 3.3.2. Effect of Stripping Agent Concentration

**Figure 6:**
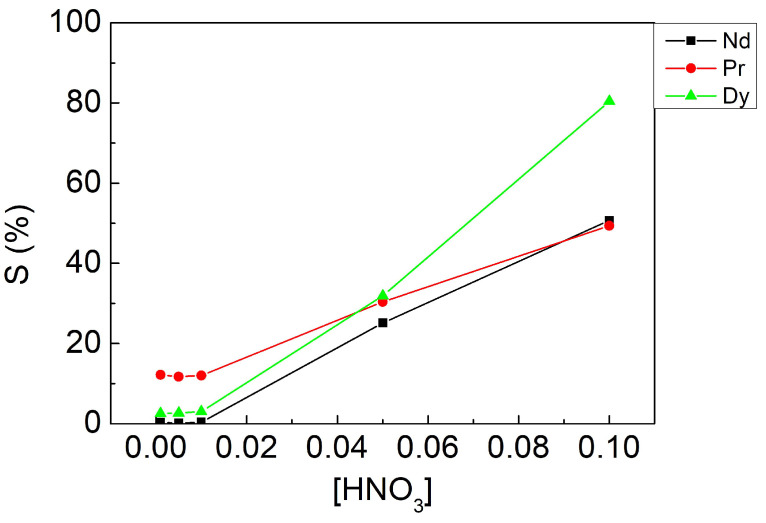
Stripping efficiencies at different nitric acid concentrations. Fixed parameters: A/O ratio of 1, contacting time of 10 min.

The concentration of nitric acid was adjusted from 0.001 to 0.1 M with fixed parameters: an A/O ratio of 1 and a contacting time of 10 min in this experiment. Figure 6 shows that stripping efficiencies were limited when the concentration was lower than 0.01 M and poor selectivity when [HNO_3_] was 0.05 M. As a result, we chose 0.1 M as the optimal parameter in view of stripping efficiencies and selectivity.

#### 3.3.3. Effect of A/O Ratio

**Figure 7:**
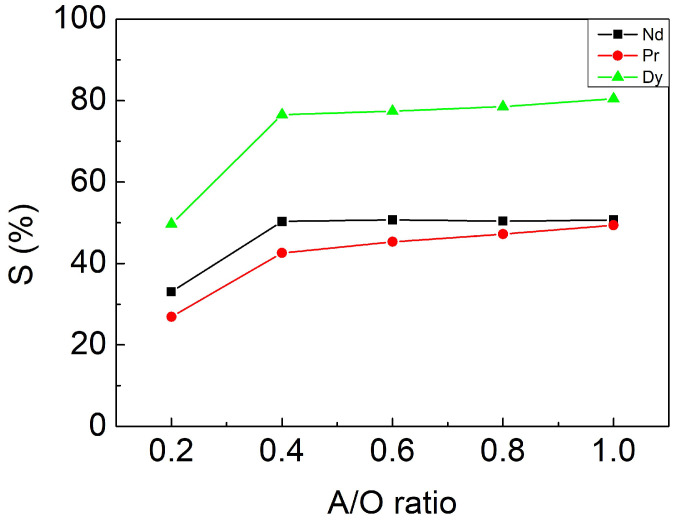
Stripping efficiencies at different A/O ratios. Fixed parameters: 0.1 M [HNO_3_], contacting time of 10 min.

For the same reason mentioned above, the aqueous to organic phase volume ratio (A/O ratio) was adjusted from 0.2 to 1 (mL/mL) with fixed parameters: 0.1 M [HNO_3_] and a contacting time of 10 min. Figure 7 shows no apparent change for stripping efficiency when the A/O ratio was higher than 0.4, where S_Dy_, S_Nd_, and S_Pr_ achieve 76%, 50%, and 42%, respectively. When the A/O ratio was 1, the best selectivity was achieved, where S_Dy_, S_Nd_, and S_Pr_ were 80.45%, 50.71%, and 49.39%, respectively. Through the one-stage extraction and stripping process, the component of Dy in the final solution accounts for 53.29% compared to the 3.55% of the initial component.

## 4. Conclusions

The results of this study demonstrate that the phosphorus ionic liquid Cyhpos IL liquid 104 could efficiently recover and separate the rare earth elements Nd, Pr, and Dy from real waste neodymium magnets through the solvent extraction process. Under the optimal experimental parameters (a contacting time of 10 min, an initial pH value of 3, 2 mM [Cyphos IL 104], and an O/A ratio of 0.6), the separation factor α_Dy/Nd_ of 45.18 and α_Dy/Pr_ of 47.93 was achieved, where E_Dy_, E_Nd_, E_Pr_ were 54.30%, 10.44%, and 9.94% respectively. For the stripping process, nitric acid was selected for the stripping agent due to the selectivity of Dy over Nd and Pr from our experimental results. A stripping efficiency of 80.45% for Dy in the final solution could be reached with 0.1 M [HNO_3_], an A/O ratio of 1, and a contacting time of 10 min. In the one-stage extraction and stripping process, the components of Dy were increased from 3.55% to 53.29%. In summary, although the multi-stage extraction and the regeneration of ionic liquid and metals from the solutions after separation still need investigation, this research shows the potential of Cyphos IL 104 for the REEs recycling from secondary resources. For future studies, this system could be completed and improved to increase its applied value and to promote the industrialization of ionic liquid in the sustainable manufacturing field.

## Figures and Tables

**Table 1:** The comparison of separation factors of REEs by some extractant.

Reference	Extractant	Diluent	α_Dy/Nd_	α_Dy/Pr_	α_Pr/Dy_	α_Pr/Gd_	α_Gd/Nd_
[15]	D2EHPA	hexane	6.8 ± 0.6	7.6 ± 0.8			
[14]	TODGA	Solvent 70	39.0 ± 1.1	51.7 ± 2.1			
[17]	Cyanex 572	kerosene	237				
[17]	P81R-Cy572	kerosene	86				
[31]	HTTA	toluene			764	1.7	
[31]	A336	[C2mim][NTf2]			82	142	
[32]	[C101][SCN]	Cyanex 923	3.43 ± 0.24				
[33]	[Cyphos 104]	kerosene					20
[33]	Cyanex 272	kerosene					4

**Table 2:** The composition of waste neodymium magnets.

Element	Fe	Nd	Pr	Dy	Co	B	Ni	Cu
Wt(%)	64.62%	20.73%	5.08%	3.55%	3.25%	1.25%	0.65%	0.87%

**Table 3:** The composition of the filtrate solution.

Element	Fe	Nd	Pr	Dy	Co	B	Ni	Cu
Wt(%)	0.62%	59.51%	14.47%	9.77%	8.96%	3.27%	2.01%	1.35%

**Table 4:** Stripping efficiency with different chemicals. Fixed parameters: concentration of 1 M, A/O ratio of 1, contacting time of 10 min.

S	Nd	Pr	Dy
HCl	41.87%	40.19%	65.28%
HNO_3_	50.37%	45.63%	81.25%
H_2_SO_4_	48.02%	43.64%	76.42%
H_2_C_2_O_4_	35.01%	35.78%	61.21%
NH_4_OH	5.29%	11.05%	1.74%
